# Pharmaceutical/food grade titanium dioxide particles are absorbed into the bloodstream of human volunteers

**DOI:** 10.1186/s12989-015-0101-9

**Published:** 2015-09-02

**Authors:** Laetitia C. Pele, Vinay Thoree, Sylvaine FA Bruggraber, Dagmar Koller, Richard PH Thompson, Miranda C. Lomer, Jonathan J. Powell

**Affiliations:** Medical Research Council Human Nutrition Research, Elsie Widdowson Laboratory, Fulbourn Road, Cambridge, CB1 9NL UK; Department of Gastroenterology, Guys and St Thomas’ NHS Foundation Trust, St Thomas’ Hospital, London, SE1 7EH UK

**Keywords:** Titanium dioxide, Particles, Absorption, Blood stream

## Abstract

**Background:**

Exposure to persistent engineered nano and micro particles via the oral route is well established. Animal studies have demonstrated that, once ingested, a small proportion of such particles translocate from the gastrointestinal tract to other tissues. Exposure to titanium dioxide is widespread via the oral route, but only one study has provided indirect evidence (total titanium analyses) of absorption into the blood stream in humans. We sought to replicate these observations and to provide additional evidence for particulate uptake.

**Findings:**

Human volunteers with normal intestinal permeability were orally administered 100 mg pharmaceutical/food grade titanium dioxide. Blood samples were collected from 0.5 to 10 h post ingestion and analysed for the presence of reflectant bodies (particles) by dark field microscopy, and for total titanium by inductively coupled plasma mass spectrometry (ICP-MS). Blood film analyses implied early absorption of particles (2 h) with a peak maximum at 6 h following ingestion. The presence of these reflectant particles in blood roughly mirrored the levels of total titanium by ICP-MS, providing good evidence for the latter being a measure of whole particle (titanium dioxide) absorption.

**Conclusions:**

This study shows that a fraction of pharmaceutical/food grade titanium dioxide is absorbed systemically by humans following ingestion. It confirms that at least two routes of particle uptake may exist in the human gut- one proximal and one distal. Further work should quantify human exposure and uptake of such persistent particles.

## Background

Oral exposure to non-biological nano- and micro- particles from the diet, environment, and man-made health and hygiene products, is now well established [1]. Persistent particles, meaning those that are not broken down easily by the gastrointestinal tract or intracellularly, have been the subject of particular attention as animal studies indicate that they may migrate from the gut mucosa to draining lymph nodes and then become translocated to most tissues of the body [2]. However, there is much less evidence in man. Examination of surgical and post-mortem specimens has confirmed that there is retention of engineered particles in the human intestinal mucosa, especially the Peyer’s patch lymphoid follicles [3–5]. Using large micron-sized biological particles (such as starch and pollen), Volkeimer has convincingly shown their appearance in the blood stream following ingestion by humans. With histological studies in rodents he developed the concept of ‘persorption’ whereby holes in the tips of intestinal villi allow the ingress of particles [6, 7]. This and other mechanisms of uptake by the gut have been studied and reviewed by us [1] and others [8, 9]. Oral absorption of particulate titanium dioxide (TiO_2_), a common food additive and excipient used in nutraceuticals, pharmaceuticals and toothpaste [10], has been especially well studied in animals [11], but barely so in man. To date, only Bockmann *et al.* have shown in humans that measurable increases in total titanium (Ti) levels occur in the blood stream following ingestion of capsules containing TiO_2_ particles [12]. Given that TiO_2_ is so resistant to dissolution, and that it accumulates in human intestinal cells [3–5], it is probable that Bockmann *et al.* were showing direct particle uptake, although this was not measured. Despite high interest on the use of TiO_2_ particles and their potential impact following acute and chronic exposure, investigative studies looking at TiO_2_ absorption in humans have only been reported once, in 2015 [13], since the initial published findings in the year 2000 [12]. However, in the recent work, high baseline values appear to have prevented observations on the appearance of Ti in the circulation following ingestion of TiO_2_ [13]. Here, in a simple pilot study that used pharmaceutical and food grade anatase TiO_2_ particles, we sought to corroborate the earlier findings of Bockmann *et al.* and, further, to confirm that the increased levels of Ti observed in blood are due to whole particle uptake into the circulation. As single particle analysis was not fully developed for quantitative analysis of blood samples, we chose to use a simple semi-quantitative method for particle detection, namely dark field microscopy. TiO_2_ readily reflects light so particles can easily be detected in complex biological samples using this technique [14]. A 100 mg TiO_2_ oral dose was chosen as this is to be at the upper end of the normal daily intake of engineered particles by humans [10], and the sampling intervals were similar to those of Bockmann *et al.* (*i.e.* between 0–10 h). We additionally determined whether blood Ti levels mirrored the presence of reflectant particles in blood as together they should provide unambiguous evidence of TiO_2_*particle* absorption.

## Methods

### Preparation of permeability solution and TiO_2_ capsules

To assess that the volunteers had normal intestinal permeability, an iso-osmolar test sugar solution (300 mmol/kg) containing 0.5 g D-xylose, 1 g L-rhamnose, 0.2 g 3-O-methyl-D-glucose and 5 g lactulose was prepared and 100 ml administered orally per subject. Gelatine capsules, containing 50 mg pharmaceutical/food grade anatase TiO_2_ (Kronos® 1171; reported manufacturer’s d50 of 260 nm, Fagron UK,) were manufactured by St Thomas’ Hospital Pharmacy (London).

### Conduct of the study

Following ethical approval (EC01/037) and informed consent, eight healthy (self-reported) volunteers were recruited; seven completed the study as blood could not be withdrawn from the cannula of one subject. First, subjects were provided with the permeability solution, 1 l of deionised water and a 2 l urine bottle containing the preservative thiomerasol. Subjects were asked not to consume any dairy products from lunchtime the day before the test. At 7.00 am following an overnight fast, subjects emptied their bladders and provided a baseline urine sample in 50 ml universal tubes. They then ingested the 100 ml test sugar solution and during the following two hours they were only allowed to drink deionised water. Urine was collected thereafter for 5 h in the 2 l urine bottles (*i.e.* until 12.00 pm).

At 9.00 am, a peripheral venous cannula was inserted and a 5 ml baseline blood sample taken. Immediately following this, subjects ingested two capsules each containing 50 mg of pharmaceutical/food grade TiO_2_ (total 100 mg) with 250 ml water - one capsule was taken immediately following the other. Blood samples were then taken at baseline (0), at 30 min, and at 1, 1.5, 2, 3, 6, 8 and 10 h post TiO_2_ ingestion. Blood samples were heparinised and analysed as described below.

Normal drinking water was provided throughout the study and meals, free from added TiO_2_, were provided at 10.00 am (Breakfast: wholemeal toast with salted butter and strawberry jam), 1.00 pm (Lunch: chicken and green pepper stir fry with boiled rice, fresh fruit and a small packet of plain crisps) and 4.00 pm (Mid-afternoon snack: chocolate biscuit and/or fresh fruit). Tea and coffee with milk and/or sugar were allowed as desired from 11 am onwards.

### Identification of TiO_2_ in blood by dark field microscopy

One drop of blood (~45 μl) from the syringe following sampling (*i.e.* non-heparinised) was placed on a clean, freshly opened glass slide. Superfrost plus slides were used to allow cell adherence. The blood was spread thinly with a clean slide to aid monolayer formation and a cover slip was then sealed in place with nail vanish to prevent sample drying. The slide was examined by light microscopy first at x 100 magnification and then x 400. Random areas were visualised at x 400 using a dark-field condenser to allow the titanium dioxide particles to be easily observed as bright white discrete particles in contrast, for example, to cell debris which was much less bright and less punctate, and generally translucent. The estimation of particles within each field was based on four reflectance grades; namely 0, 1, 2 and 3, and the operator was blinded to sample codes. A reflectance grade of 0 indicated 5 or less particles/field, a grade of 1 showed 5 to 10 particles/field, a grade of 2 showed 10 to 20 particles/field, and a grade of 3 indicated > 20 particles/field. Reflectance grades, rather than absolute numbers, were used for rapid data acquisition and because of the semi-quantitative nature of detection. Visualisation of TiO_2_ particles by dark field microscopy could only be carried out in five out of seven subjects due to clotting of blood in two subjects.

### Measurement of Ti by Inductively Coupled Plasma- Mass Spectrometry (ICP-MS)

Aliquots of collected heparinised blood samples were acid digested using sub-boiling nitric acid diluted with Milli-Q water and high-purity hydrogen peroxide [15]. Total Ti concentrations were determined by High-Resolution ICP-MS at the Department of Physical and Analytical Chemistry, University of Oviedo, Spain, using an Element 2 (Thermo Fisher Scientific, Waltham, MA, USA) working at medium resolution (RS = 4000) to analyse the isotope of ^47^Ti [15, 16]. For quantification, the method of isotope dilution was applied [17]. Measurement of Ti was carried for time points 0–10 h except in two subjects where samples at 8 h in both, and 10 h in one, could not be collected.

### Statistics

The estimation of particle numbers present in blood films and the levels of Ti following ingestion of TiO_2_ were assessed using a two-tailed paired t–test. The relationship between reflectance grades (*i.e.* TiO_2_ particles within blood) and the levels of Ti measured by ICP-MS was determined using a Spearman correlation test. For all tests, significance was taken at p < 0.05.

## Results and discussion

**Fig. 1:**
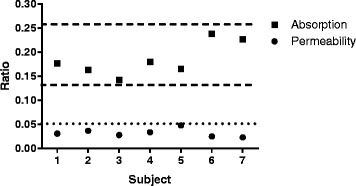
Intestinal permeability and absorption. Small molecule permeability (black circles; lactulose:rhamnose excretion) and absorption (black squares; rhamnose:3-O-methyl glucose excretion) ratios. The reference ranges for permeability (dotted line) and absorption (dashed line) are ratios of <0.05, and 0.132 – 0.258, respectively

**Fig. 2:**
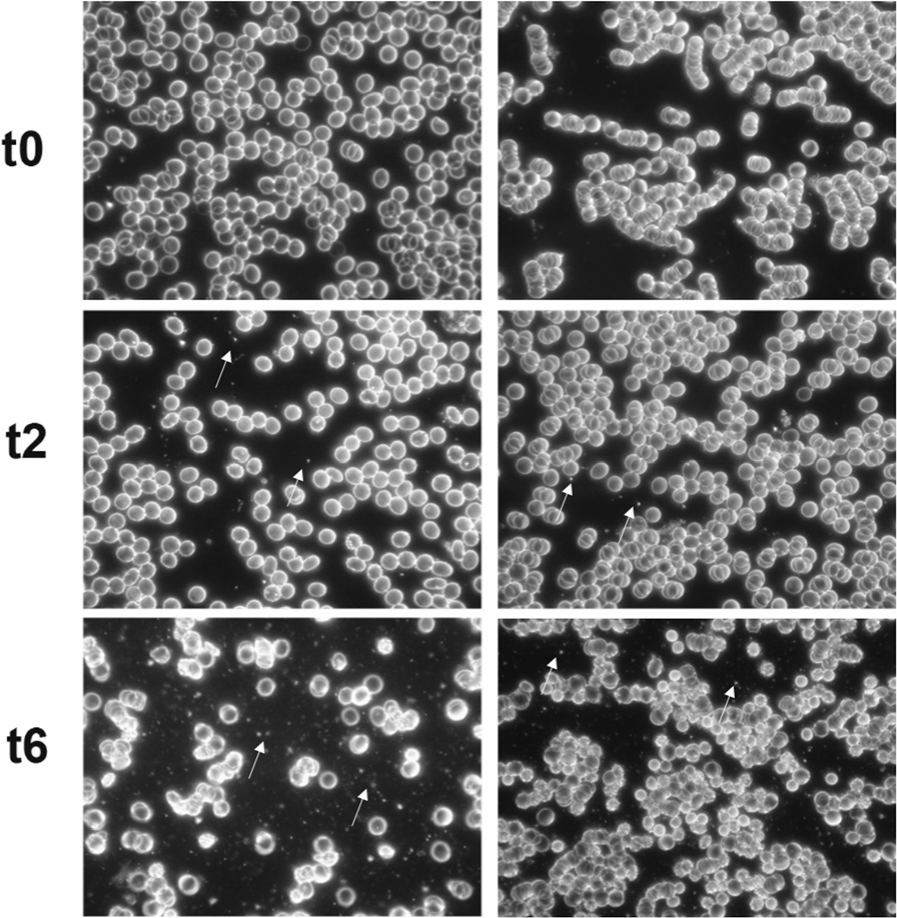
Reflectance micrographs of blood smears. Representative examples of visualisation of blood smears at x 400 magnification by dark field microscopy at baseline (t0), 2 h (t2) and 6 h (t6) following ingestion of 100 mg TiO_2_ capsules, in two subjects. Examples of particle-positive signals are indicated by the white arrows. A few particles were present at t0, but were always below 5 particles per field of analysis. Data for all five subjects are displayed and summarised in Fig. 3

**Fig. 3:**
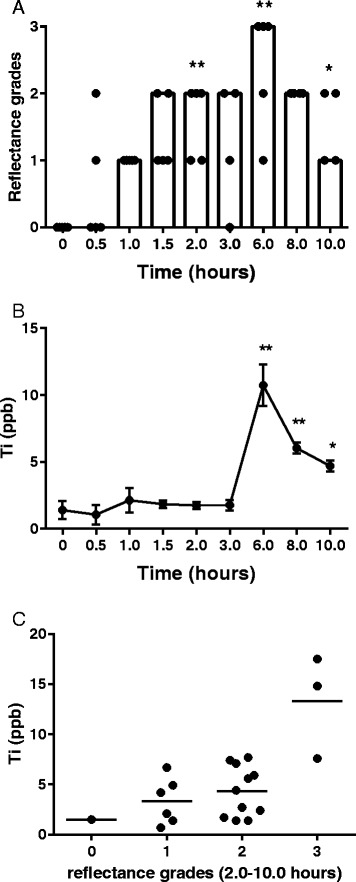
Absorption of TiO_2_ particles into the blood stream. **a**- Grading of the frequency of discrete particulate reflectance in blood smears by dark field microscopy following ingestion of 100 mg TiO_2_ capsules (n = 5 subjects). Data are plotted as mode (column) and individual grades (closed circles). * *p* < 0.05 and **p < 0.01 vs time point 0, two-tailed paired *t* test. **b**- Total Ti levels (ppb) measured by ICP-MS in the same blood samples as above with two further subjects (total seven). Data are represented as mean +/− SEM. *p < 0.05 and **p < 0.01 vs time point 0, two-tailed paired *t* test. **c**- Correlation between reflectance grades and total Ti levels in blood for the five complete subjects only. Pearson correlation *p* = 0.0058 and *r* = 0.5803

The seven subjects had normal gut permeability and absorption characteristics of the different sugars based upon their urinary excretion data (Fig. 1). Some of the ingested TiO_2_ was absorbed directly into the blood stream in its particulate form based upon dark field microscopy (Figs. 2 and 3a). Positive signals were assumed to be TiO_2_ particles since no other sources of particles were provided (*i.e.* the subjects only ingested TiO_2_ particles and the lunch provided had no particulate additives). As such, whilst occasional positive signals were noted at baseline (*i.e.* bright discrete particles), observations always numbered less than 5 per field (*i.e.* scored 0; Figs. 2 and 3a). Significant increases in positive signals were observed in the blood films from 2 h onwards (Figs. 2 and 3a, *p* < 0.05). Dark field microscopy findings were roughly mirrored by ICP-MS measurements of total Ti within the same blood samples (Fig. 3b, c, t2-t10), with both techniques demonstrating a peak of absorption at 6 h post ingestion (Fig. 3a, b; *p* < 0.05). Indeed, there was a positive correlation between reflectance grades and total Ti levels (Fig. 3c, *p* = 0.0058 and *r* = 0.5803).

Our current findings substantiate those of Bockmann *et al.,* in which a comparable trial design demonstrated a similar pattern of titanium loading into the blood stream of human subjects following the ingestion of TiO_2_ [12]. However, we now show that these total Ti signals by ICP can be reasonably attributed to the actual absorption of whole particles (*i.e.* TiO_2_). Confirmation that the direct uptake of particulates occurs early (Figs. 2 and 3a), and peaks late (Fig. 3a), has some important implications for understanding oral particle exposure to humans. First, it confirms that persistent particles (*i.e.* those that evade gut and cellular digestion) can be taken up systemically in man, confirming the results of many animal studies and models of uptake [18–20]. Secondly, it provides further evidence to agree with Bockmann’s observations that, in humans, a bimodal pattern of particle uptake occurs: it starts early (*i.e.* visible by 2 h following ingestion) but peaks later (*i.e.* 6 h following ingestion). If so, this pattern may be explained by some absorption in the proximal small intestine (duodenum/jejunum) [1, 7] with later Peyer’s patch uptake in the more distal small intestine (ileum) [1]. Peyer’s patches are intestinal lymphoid follicles lined with specialised microfold (M) cells that specifically and avidly capture small particles. How such non-biological particles are then distributed is not clear, although local cellular uptake of the particles may occur (*i.e.* in mucosal antigen presenting cells [21]), with the potential for translocation to the mesenteric lymph nodes and then beyond, as well as distribution to other organs as shown in animal studies. For the latter, blood-borne transport must occur, as we confirm, although whether the particles are transported within or outside of immune cells requires confirmation.

The route of putative proximal absorption is less clear. As noted earlier, the term persorption has been coined by- and the phenomenon well described by- Volkeimer [7]. Additionally, the sporadic occurrence of small M-cell rich areas throughout the small intestine [22] may also provide ports of entry for particles, or the nano fraction of the ingested particles could pass through regular epithelial cells to underlying dendritic cells, as recently described [23]. It was not our intention to size the absorbed TiO_2_ particles and hence we did not measure the size distribution of the TiO_2_ administered. Nevertheless, a recent publication investigating the same TiO_2_ particles quotes a primary particle size distribution of 88.9-200.6 nm (d10-d84) [24] which is smaller than the stated manufacturer's d50 of 260 nm, perhaps because the latter also measured small agglomerates during analysis. More importantly the particles represent a ‘real life’ scenario of what humans can be exposed to, either in food or pharmaceuticals, and clearly a fraction of it will be nano-sized [11]. Further work will need to confirm the proposed proximal intestinal uptake of particles, in humans, and the underlying mechanisms, but a preference for penetration by the nano-sized fraction should not be ruled out as this is seen in rodents [18, 19].

Finally, although the ingestion of 100 mg TiO_2_ exceeds human intestinal exposure as a single dose, it falls within daily limits [10] and higher dosing facilitates analytical detection. Therefore, whilst absolute particle absorption may be lower in the general population than observed here, the percentage absorbed is likely to be higher. Indeed, Powell and colleagues have estimated that adult humans may absorb 10^12^ TiO_2_ particles/person/day [25]. Further quantification work of titanium dioxide absorption at different doses, and translation to particle numbers and sizes is now needed.

The recent implementation of new software for single particle ICP-MS analysis [26], advancements of hyphenated techniques like field flow fractionation [27] and hydrodynamic chromatography [28] combined with ICP-MS and the emergence of a triple quadrupole ICP-MS [29] should all aid the development of quantitative techniques to help assess human exposure to TiO_2_.

In summary, we show here that a portion of ingested pharmaceutical and food grade TiO_2,_ to which humans are very frequently orally exposed, is directly absorbed, as particles, into the blood stream of healthy volunteers. The seemingly early absorption and late peak of uptake point towards two distinct periods and perhaps routes of uptake: one early in the proximal small intestine and one late in the distal small intestine. Quantitative measurements are now merited to estimate real human exposures.

## Abbreviations

ICP-MS: Inductively coupled plasma-mass spectrometry
Ti: Titanium
TiO_2_: Titanium dioxide
